# Expectations and psychological issues before genetic counseling: analysis of distress determinant factors

**DOI:** 10.1186/s13053-020-00142-1

**Published:** 2020-04-29

**Authors:** Zelmira Ballatore, Raffaella Bracci, Elena Maccaroni, Lucia Svarca, Francesca Bianchi, Laura Belvederesi, Cristiana Bruciati, Silvia Pagliaretta, Alberto Murrone, Federica Bini, Mirco Pistelli, Giulia Ricci, Rossana Berardi

**Affiliations:** 1 Clinica Oncologica, Azienda Ospedaliero Universitaria Ospedali Riuniti di Ancona Umberto I G M Lancisi G Salesi, Ancona, Italy; 2 Oncologia, Azienda Ospedaliera Ospedali Riuniti Marche Nord, Presidio Santa Croce, Fano, Italy; 3 Neuropsichiatria Infantile, Azienda Ospedaliero Universitaria Ospedali Riuniti di Ancona Umberto I G M Lancisi G Salesi, Ancona, Italy

**Keywords:** Genetic counseling, HNPCC, HBOC, Psychological distress, Genetic test

## Abstract

**Background:**

Hereditary non-polyposis colorectal cancer (HNPCC) and Hereditary Breast and Ovarian Cancer Syndrome (HBOC) are the most common hereditary cancer syndromes in which a genetic test is available. Potential risks associated with testing include psychological harm, emotional distress and insurance problems.

**Methods:**

The aim of the present study is to investigate determinants of distress in a sample of Italian subjects undergoing genetic counseling. Demographic information and psychological distress were assessed by using a self-reported questionnaire and the “Hospital Anxiety and Depression Scale” (HAD), before attending the first counseling session.

**Results:**

Of the all subjects referred for the first time to our Center (January 2012–June 2013), a total of 227 were eligible (female/male = 174/53) for the survey, 134 (59%) were oncologic patients and of these, 116 received genetic test (36 for HNPCC and 80 for HBOC). The remaining 93 (41%) were healthy subjects referred for suspected familiar history and of this group, 65 subjects performed predictive test in a family with a known pathogenic mutation (53 for HBOC and 12 for HNPCC). Affected subjects had a significantly higher level of anxiety (*p* = 0.02) and HAD global score (*p* = 0.01) than healthy ones. There was no difference in HAD score between individuals testing for different syndromes (*p* = 0.3). In the affected subgroup, there was a significant linear correlation between the HAD anxiety score and how much subjects perceived their disease as hereditary (*p* = 0.01). Female and younger subjects had higher levels of anxiety (*p* = 0.05). Also healthy single subjects show more general distress (*p* = 0.02) than those with a partner.

**Conclusions:**

Greater level of distress identified on females, single and younger subjects.

## Background

In about 5–10% of breast or colorectal cancer patients, the onset to the disease is the result of a heritable mutation (germline mutation), in a cancer predisposition gene. At-risk individuals tend to develop benign and/or malignant tumors at earlier age than usual and they have increased risk of developing more than one primary tumor.

In addition, the siblings and offspring of an affected patient have a 50% chance of inheriting the cancer-predisposing mutation segregating in the family, consistent in most cases with autosomal dominant inheritance [1].

Actually genetic testing is available for several cancer predisposition genes and became part of clinical practice. Familial Adenomatous Polyposis (FAP), Hereditary non-polyposis colorectal cancer (HNPCC), also known as Lynch Syndrome, and Hereditary Breast and Ovarian Cancer Syndrome (HBOC) are the most prevalent and investigated hereditary cancer syndromes [2, 3].

Genetic testing is always supported by counseling, which consists of pedigree reconstruction, risk assessment and education, facilitation to genetic testing [4, 5]*.* The psychological impact of genetic susceptibility testing for hereditary cancer is reported in the literature over the last two decades [2, 6–11]. Risks associated with genetic testing include emotional distress, psychological harm and potential insurance and employment discrimination [12].

In fact, hereditary cancer genetic testing has relevance for the entire family system: relatives with mutation may become aware of having increased cancer risk whilst those non-carriers may feel responsible or guilty [3, 13]*.* Furthermore, any previous cancer experience could greatly influence individual risk perception, emotional state before testing and experience of coping with a positive test result [3]. Literature data showed that presence of pre-existing psychological distress and concerns are the best know psychological prognostic factors [14]. This is one of the reasons why most individuals apply for genetic counseling on their own, having high self-perceived risk of genetic predisposition [15–18]. In a review article by Gopie et al. (2012), it appeared that young age, perception of high risk, pre-existing psychological distress, a passive way of coping, little social support and family members with cancer were predictive of psychological problems and/or reduced quality of life. Counselees who were single were depressed [19] and the type of cancer syndrome (HBOC or Lynch syndrome) had no influence [3]. Aim of our study was to investigate distress in Italian subjects referred for a suspected cancer syndrome before performing genetic counseling and evaluating anxiety and depression level though the validated HAD scale.

## Methods

### Participants

In this cross-sectional study, we collected data from Italian subjects referred for counselling to the Genetic Oncology Center of the Marche Region, from January 2012 to June 2013. Participants were self-referred, referred by a relative or by their general physician or by specialist (oncologist, gynecologist, gastroenterologist). They were healthy subjects or cancer patients. Selection criteria were: age of 18 years or older, no previous genetic counseling/test, no diagnosis of psychiatric disorders, ability to give informed consent.

### Socio-demographic data

Age, sex, marital status, education level, having children, lifestyle and habits were obtained through specific items in the questionnaire. Data about personal and relatives medical history were collected by the counsellor and recorded in the medical source documents.

### Questionnaire

Prior to attending the genetic counseling sessions, all subjects were provided with verbal information about the study and asked for consent in writing, which they all did provide. Besides socio-demographic data, the questionnaire was made of several multi-choice items regarding attitudes towards and reasons for pursuing genetic test.

### HAD questionnaire

Psychological distress was assessed by a self-reported questionnaire. This was administered pre-counseling intervention that included the Hospital Anxiety and Depression Scale (HADs). The HADS is a fourteen item scale that generates ordinal data. Seven of the items relate to anxiety and seven relate to depression. Each item has a choice of four answers with scores ranging from 0 (no distress) to 3 (maximum distress). The subscales score range from 0 to 21. On either subscale, scores 0–7 are considered “normal”, scores 8–10 represent “borderline” and scores of 11 or more represent a “pathological” situation [20]. The HADS has been validated for use in Italy [21].

Zigmond and Snaith created this outcome measure specifically to avoid reliance on aspects of these conditions that are also common somatic symptoms of illness, for example fatigue and insomnia or hypersomnia [20].

### Statistical analysis

Variance analysis and Chi square test were used to evaluate sociodemographic and clinical features.

Fisher exact tests were used to analyze demographic and clinical characteristics and HADs findings to estimate association between categorical variables. Linear regression was used to study continuous variables. A statistic significant level of 0.05 was chosen. Statistical analysis were performed with STATA MP.11.0 software.

## Results

### Participants

The survey was proposed to a total of 250 subjects and of these, 227 partially or totally completed the questionnaire before the first genetic counseling interview. There were 23 drop out patients and the reason was the reported absence of interest.

The descriptive analysis of the sample is described in Tables 1 and 2. The *P* values are referring to first entry among the three variables The subjects were attending the Cancer Genetic Center both for HBOC and suspected hereditary colon cancer syndromes. The mean age of the counselees was 48.7 years (range18–83 years), 53 subjects were males, 174 were women. The majority are patients with a previous cancer (134 affected, 59%), 93 were healthy subjects referred because of being part of a high risk family.

**Table 1:** Socio-demographic characteristics of all sample and Chi Square Analyses

Features	All subj (%)	Affected subj (%)	Healthy subj (%)
**Age range (years)**	18–83	19–83	18–74
**Mean (± s.d.)**	48.7 ± 0.9	53.1 ± 1.1	42.5 ± 1.3
**Sex**			
female	174	110 (82.1)	64 (68.8)
male	53	24 (17.9)	29 (31.2)
**Having Children**			
yes	170	111 (82.8)	59 (64.1)
no	57	23 (17.2)	33 (35.9)
**Marital Status**			
single	35 (15.6)	13 (9.8)	22 (23.7)
separated/divorced/widowed	26 (11.5)	18 (13.6)	8 (8.6)
married/cohabiting	164 (72.9)	101 (76.5)	63 (67.7)
**Education Level**			
≥ high school	153 (67.4)	87 (65.4)	65 (70.6)
< high school	74 (32.6)	46 (34.6)	27 (29.4)

**Table 2:** Investigative questionnaire and participants answers. Chi Square analyses as appropriate

MULTIPLE CHOICE QUESTION	All subj (%)	Affected subj (%)	Healthy subj (%)	p
**1.Having received information about genetic counseling from:**				
health care professionals	137 (61.4)	100 (74.6)	37 (39.8)	< 0.001
other sources	87 (38.3)	31 (23.1)	56 (60.2)	
missing data	3 (1.3)	3 (2.3)	0 (0)	
**2.The initiative of deciding to apply for the test as to be taken:**				
in agreement with the oncologist/health care physicians/familial member	110 (48.5)	78 (58.2)	32 (34.4)	< 0.001
on their own	107 (47.1)	50 (37.3)	57 (61.3)	
missing data	10 (4.4)	6 (4.5)	4 (4.3)	
**3.How do you think of your family relationship?**				
very good	118 (52.0)	69 (51.5)	49 (52.7)	0.29
good	91 (40.1)	53 (39.5)	38 (40.9)	
problematic/bad	13 (5.7)	8 (6.0)	3 (3.2)	
missing data	5 (2.2)	4 (3.0)	3 (3.2)	
**4.Do you think your lifestyle could have affected your health?**^a^				
yes		28 (20.9)		
no		105 (78.3)		
missing data		1 (0.8)		
**5.Do you think your disease is hereditary?**^a^				
yes		35 (26.1)		
no		6 (4.5)		
i don’t know		90 (67.2)		
missing data		3 (2.2)		
**6.How much do you perceive as hereditary your disease ranging from 0 to 10?**^a^				
Mean (± s.d.)		6.9 ± 0.2		
**7.Are you at risk of an hereditary diseae?**^b^				
yes		58 (60.4)		
no		26 (27.1)		
i don't know		0		
missing data		12 (12.5)		
**8.How much do you perceive your risk of an hereditary condition ranging from 0 to 10?**^b^				
Mean (± s.d.)		7.2 ± 0.3		

^a^ only for affected subjects. ^b^only for predictive test

Of all patients, 116 (64.1%) had a cancer medical history which was highly suspected for hereditary tumors, specifically 36 patients suspected for HNPCC and 80 patients for HBOC syndromes. A further 65 (35.9%) conselees were healthy subjects referred for a possible predictive test. The remnant were referral patient and healthy subjects without a highly influential family tree.

### Psychological distress

We measured a significant difference in cumulative HAD score between affected subjects and healthy individuals, the first ones presented higher distress at a borderline and/or pathological level if compared to healthy participants (*p* = 0.03). This result was not confirmed on the single scales of anxiety and depression, respectively, but there was the evidence of a trend in both scales, so subjects with cancer resulted generally more stressed than healthy ones (predictive test) (*p* = 0.08). Women reported a trend towards higher distress (*p* = 0.05) than men, while there were no significant differences based on age and marital status. However, there was no difference in HAD score between individuals testing for different syndromes (*p* = 0.3). In the predictive test subgroup, single subjects presented higher level of distress (*p* = 0.04) than those who had a partner, on the contrary there was no evidence of this trend in the affected probands subset. Having children seemed to correspond to a higher score of anxiety and general distress (respectively *p* = 0.09, *p* = 0.08) and in the predictive test subgroup, it was associated with a pathological level of anxiety (*p* = 0.02); this result was not present in the affected subgroup.

Of 227 participants, 137 (61.2%, 100 with cancer and 37 healthy subjects) received information about genetic counseling from health care professionals (physician, oncologist, radiotherapist etc), the remaining 87 (38.2%, 56 healthy and 31 affected subjects) received information from other sources (for example affected relatives, web, newspapers, television etc**).** Most of healthy subjects stated that the decision to undergo predictive testing had to be taken principally on their own, while patients with cancer considered genetic testing only in agreement with the oncologist/health care physician. This association resulted statistically significant (*p* < 0.01). Those who had received information about genetic counseling by health care professionals were more frequently subjects with cancer (74,6%), and they show a higher education (65,4%) and a higher level of HAD score than those who received information from other sources.

Among the 131 patients with cancer, 35 answered positively to the question “do you think your disease is hereditary?”, while 90 participants responded they did not know. One hundred twenty two answered to the question “how much do you perceive your disease as hereditary ranging from 0 to 10?” and the mean value of the answers was 6.9 ± 0.2. Interestingly there was a significant linear correlation between the HAD anxiety score and how much subjects perceived their disease as hereditary (*p* = 0.01).

In 58 healthy subjects perceived to be at risk of a hereditary disease and 49 (72% of the sample) answered to the question “how much do you perceive your risk of a hereditary condition ranging from 0 to 10?” and the mean value of the answers was 7.2 ± 0.3. Also in this group there was a significant linear correlation between the HAD anxiety score and how much the risk was perceived as hereditary (*p* = 0.04).

Regarding family relationship, those healthy subjects who thought their family relationships were bad had a higher level of anxiety then those who perceived them good (*p* = 0.01).

## Discussion

In most cases, the perceived likelihood for carrying a gene mutation decreased after testing and subjects referred for genetic counseling found the counseling process extremely helpful for future medical decision-making [15–18]. Butow et al. concluded that genetic counseling is optimal in improving accuracy of cancer risk perception since many subjects largely overestimate it [22]*.* Furthermore, evidence from systematic reviews illustrates that a genetic counseling intervention does not appear to increase distress and therefore could improve the accuracy of individual’s perceptions of their personal risk [23, 24]*.*

Our results show that cancer patients seem to report a higher level of distress and worries than disease free subjects.

It could be hypothesized that the diagnosis of a neoplasm often leads to psychological instability, although previous studies are equivocal about the effects of a cancer diagnosis on distress [25–27]. However recent data also shows that patients having genetic testing shortly after a diagnosis develop a cancer-related distress due to a significantly different number of psycho-emotional symptoms, which decreased with time [28, 29].

Maybe healthy subjects with an affected first degree relative may have already accepted that cancer runs in their family and this may have reduced the probability of experiencing a high anxiety level. In addition, we confirmed that subjects with higher distress were more frequently women and more often single, without an adequate social support or with bad family relationships [15, 30–32]. Although this finding has already been reported, its explanation has not been fully established, the most presumable hypothesis is that a good familial, social and emotional support promotes a better adaptation to stressful events such the genetic counseling process.

Healthy subjects appeared less distressed, but those with children presented higher levels of anxiety than those childless. This is reasonable considering that disease free members of an at-risk family could experience greater distress because of the consequently increased risk for their children. Most of these subjects experienced a lot of suffering in their family as children or siblings of cancer patients and this suffering quite often modeled their infancy or adolescence [33].

High risk subjects seem to consider their lifestyle and their habits irrelevant in a sort of “fatalistic” view of their personal genetic predisposition to cancer.

We confirmed greater adherence to genetic testing on the advice of their physician. So that, for adequate referral, physicians should be fully aware of the criteria of genetic risk and the process of genetic counseling and testing [18, 34].

Moreover, our results showed a positive correlation between HAD anxiety score and the perceived risk of hereditary predisposition “per se” both in cancer patients and in healthy individuals referred for a predictive test. Even if most subjects in both subgroups were not able to answer with certainty about the risk of inheritance, the majority of them reported they perceived it to be high.

It is likely that due to an overestimation of the personal and familial hereditary risk the HAD anxiety score could be influenced by the uncertainty of the personal genetic predisposition. This result is probably due to the lack of information necessary to give a real perception of risk in the pre-counseling phase. It would be interesting to assess these items in a post-counseling phase. Our survey confirms other literature results about disparities in health care access, indeed highly educated subjects more often received information about the test and hereditary risk of cancer from health care professionals [30]. However, education level does not seem to impact on distress. Access to test should be available also in less educated individuals. The strengths of this study are its prospective design, the homogenous population and the large study sample, conversely one of the major limitation is that the study investigates only pre-test consultation and has a limited follow up. Most of the participants had been appropriately referred to genetic counseling, in fact 79.7% of them were eligible for DNA testing for their familial history of cancer indicating they were, actually at an increased risk. Furthermore, it is important to take into consideration that the self-reported information and that HAD’ scale, although largely used in genetic counseling for hereditary tumors, reveal a type of “general” psychological distress linked to a pathological event rather than a “cancer-specific” distress [25].

## Conclusions

This study adds to the understanding of the variables that can be used to identify subjects at higher risk of psychological distress, in order to give them appropriate strategy of coping. Investigating if the subject had cancer before, if he/she has a partner or children or lives alone, can give some information on the possible distress perceived by the counselee and give the counselor some hints on how to set up the counseling phase in order to offer the best of support, especially to counselees with more worries or emotional distress. Further investigation also in the post-test counseling phase are warranted to reach more reliable conclusions.

## Data Availability

All data generated or analysed during this study are included in this published article (and its supplementary information files).

## Abbreviations

HNPCC: Hereditary non-polyposis colorectal cancer
HBOC: Hereditary breast and ovarian cancer syndrome
HAD: Hospital anxiety and depression scale
FAP: Familial adenomatous polyposis
