# Correction to: Expectations and psychological issues before genetic counseling: analysis of distress determinant factors

**DOI:** 10.1186/s13053-020-00143-0

**Published:** 2020-05-19

**Authors:** Zelmira Ballatore, Raffaella Bracci, Elena Maccaroni, Lucia Svarca, Francesca Bianchi, Laura Belvederesi, Cristiana Brugiati, Silvia Pagliaretta, Alberto Murrone, Federica Bini, Mirco Pistelli, Giulia Ricci, Rossana Berardi

**Affiliations:** 1 Clinica Oncologica, Azienda Ospedaliero Universitaria Ospedali Riuniti di Ancona Umberto I G M Lancisi G Salesi, Ancona, Italy; 2 Oncologia, Azienda Ospedaliera Ospedali Riuniti Marche Nord, Presidio Santa Croce, Fano, Italy; 3 Neuropsichiatria Infantile, Azienda Ospedaliero Universitaria Ospedali Riuniti di Ancona Umberto I G M Lancisi G Salesi, Ancona, Italy

**Correction to: Hered Cancer Clin Pract 2020, 18: 10**

**https://doi.org/10.1186/s13053-020-00142-1**

Following publication of the original article [1], the authors identified an error in the author name of Cristiana Brugiati.

The incorrect author name is: Cristiana Bruciati

The correct author name is: Cristiana Brugiati

The author group has been updated above and the original article [1] has been corrected.

## References

[1] Ballatore Z, Bracci R, Maccaroni E (2020). Expectations and psychological issues before genetic counseling: analysis of distress determinant factors. Hered Cancer Clin Pract.

